# Rehabilitation of an Atrophied Upper Maxillary Arch With an Implant-Supported Hybrid Prosthesis

**DOI:** 10.7759/cureus.69494

**Published:** 2024-09-15

**Authors:** Ispita Roy, Kaushik Kumar Pandey, Pratibha Katiyar, Fauzia Tarannum, Shaily Tyagi, Ragini Singh, Manjari Shrivastava, Sana Khan

**Affiliations:** 1 Prosthodontics, Career Post Graduate Institute of Dental Sciences and Hospital, Lucknow, IND

**Keywords:** atrophic maxilla, dental implants, hybrid prosthesis, implant supported fixed prosthesis, intra-arch distance

## Abstract

In a completely edentulous situation, providing a fixed hybrid prosthesis with the support of dental implants could be one of the viable treatment options. Such type of hybrid prosthesis is fabricated by applying the heat-cured polymerized resin over the metal framework and screwing the complete assembly into the implants. The objective of this case report is to illustrate the rehabilitation of the form, function, and esthetics of an atrophied upper maxillary arch by utilizing an implant-supported hybrid prosthesis.

## Introduction

Dental implants are becoming more essential in dental restoration, with the high success rate of implant-supported prosthesis enhancing the esthetic expectations of both patients and clinicians [1]. Achieving satisfactory functional and esthetic outcomes requires both successful osseointegration and precise placement of fixtures designed for holding the desired prosthesis. One of the prime requisites of all prostheses supported by dental implants is to eliminate the need for full removable dentures through the placement of a fixed prosthesis supported by an implant. Only a few implants are placed for increasing the retentive properties of implant-supported overdenture [1].

All prostheses supported by dental implants can be made by two methods: ceramic bonded over cast metal framework and acrylic bonded over cast metal framework (hybrid prosthesis) [2]. The fixed hybrid prosthesis was developed to solve issues associated with unstable and least retentive removable complete dentures. During the diagnosis and treatment planning, adequate inter-arch space should be required for the success of a hybrid prosthesis. Additionally, it is crucial to evaluate other clinically relevant patient parameters, such as lip support, a high maxillary lip line while smiling, a low mandibular lip line during speech, and the patient's elevated esthetic expectations [2]. Hybrid prostheses offer numerous advantages, such as minimizing the occlusal impact during functional movement, less manufacturing cost, and high natural appearance for patients [1,2].

Additionally, a combination of tilted and axially placed implants can be effectively used in cases of partial edentulism in the posterior region of resorbed maxillae [3]. Despite the positive long-term results seen in prosthetic rehabilitation with implants, there are frequent occurrences of biological and mechanical issues. Complications during implant placement surgery, soft and hard tissue problems around implants, difficulty in mastication/speech, and less desired esthetic results may occur with a hybrid prosthesis. To avoid these complications, there should be proper diagnosis and treatment planning with an interdisciplinary approach and good teamwork along with the surgeon's skill, which plays a key role in the success of the implants. The patient's comorbid conditions, deleterious habits, such as bruxism and smoking, periodontal disease, and maintenance practices should be considered during the planning phase for hybrid prosthesis [3], highlighting the importance of teamwork and prosthetic consent for inter-arch space, type of occlusion, implant site, and angulations with the number of implants [3].

This case report elucidates the step-by-step treatment approach and desired result achieved in addressing a patient's biological and technical challenges within implant therapy, accomplished through the application of an implant-supported hybrid prosthesis.

## Case presentation

**Figure 1:**
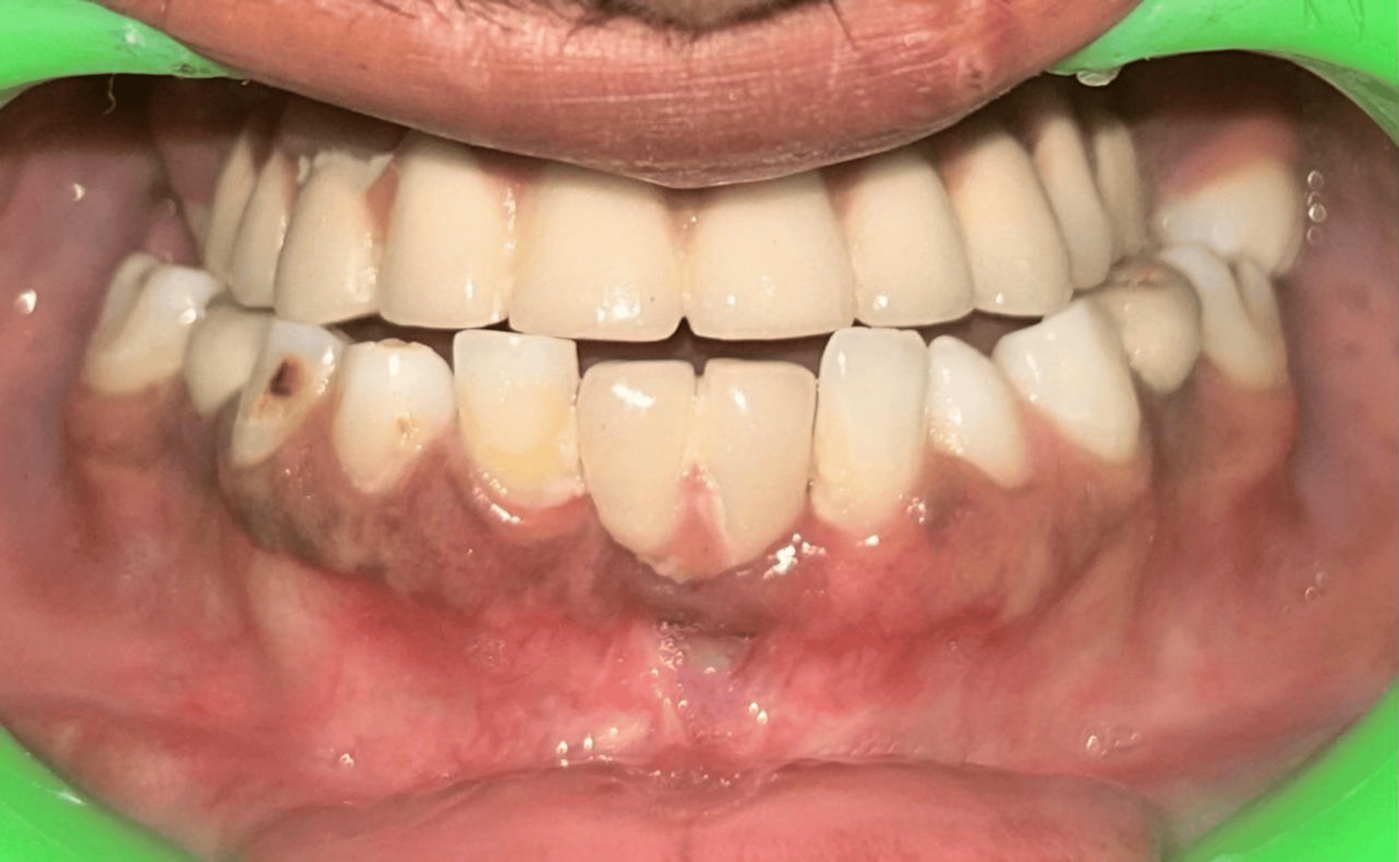
Preoperative intraoral picture showing an unaesthetic appearance due to a faulty prosthesis

**Figure 2:**
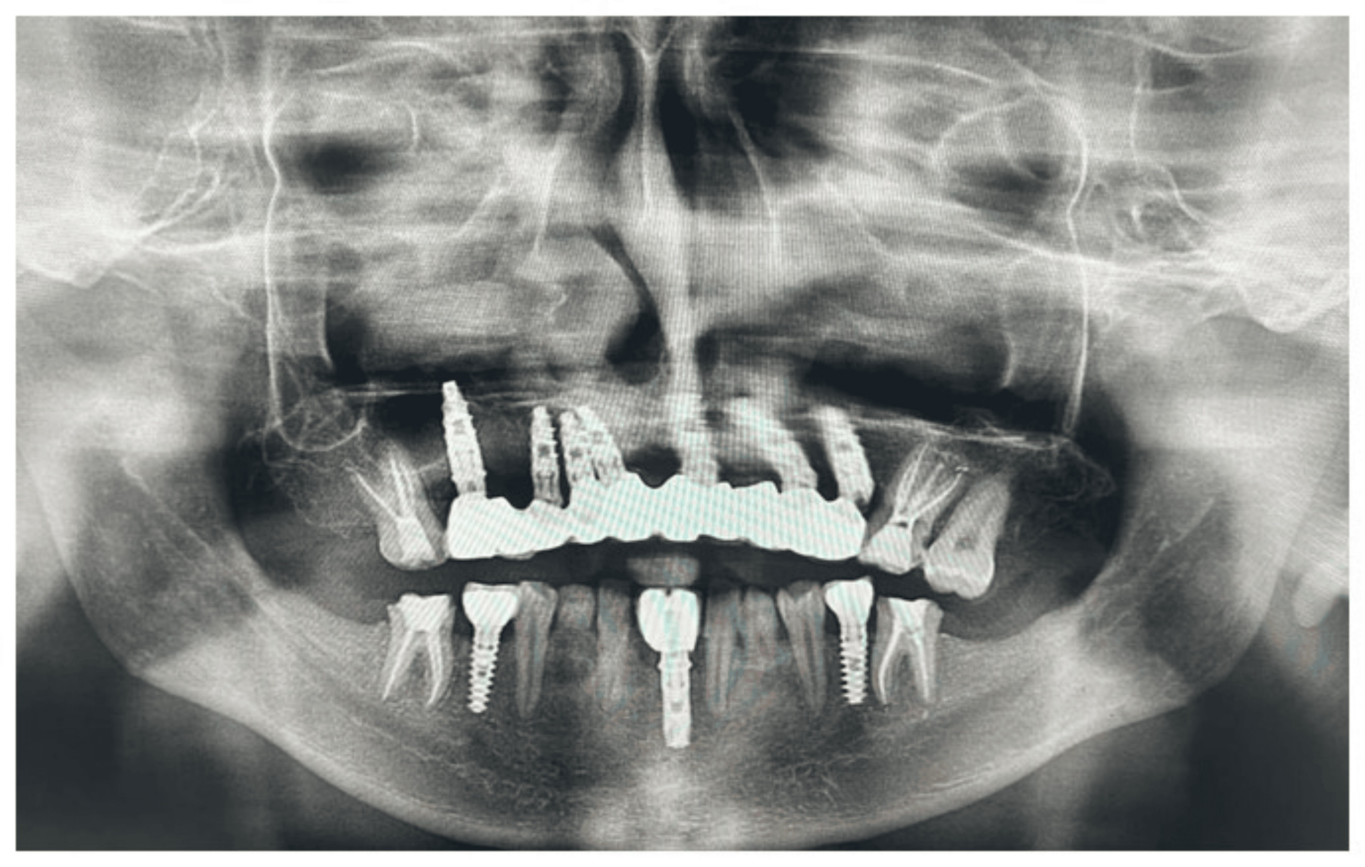
Preoperative radiograph (OPG) OPG: orthopantomography

A 26-year-old male patient presented with the chief complaint of a compromised esthetic and masticatory function. Medical history revealed that the patient had a cleft palate and undergone surgery for the same. Past dental history revealed that the patient had undergone seven implant placements in his upper maxillary arch and three implants placed in the lower mandibular arch in sites 11, 12, 13, 15, 31, 36, 46, 22, 24, and 25, which was rehabilitated with an implant-supported cement-retained fixed prosthesis five years back. Root canal-treated teeth with crowns were present in sites 16, 26, 27, 36, and 46. In this patient, mandibular growth over time was not compensated with a proportional maxillary growth and resulted in a crossbite. Clinical examination revealed a crossbite in the anterior as well as in the posterior region of the jaw, loss of facial fullness, unaesthetic appearance, temporomandibular joint (TMJ) clicking, and improper occlusion (Figure 1). Each implant site was meticulously evaluated for the condition of the peri-implant soft tissues, revealing an absence of inflammation, redness, swelling, or any signs of exudate or suppuration. Furthermore, radiographic assessment showed no evidence of inflammatory changes. It was found that all seven implants were well osseointegrated without any remarkable bone loss. The radiographic evaluation revealed an ill-fitted implant-supported prosthesis and root canal-treated teeth present in sites 16 and 26 (Figure 2). In the mandibular arch, 32 and 42 were supra-erupted, and also 34, 44, 36, and 46 were lingually tilted.

Finally, after discussing all treatment options, the patient was ready for the replacement of a maxillary implant-supported and tooth-supported fixed dental prosthesis (FDP). In the mandibular arch, only slight enameloplasty was done for the plane correction, and no further changes were made due to financial constraints. A tentative jaw record was done on the faulty prosthesis to check the loss of vertical height and found a 5 mm freeway space. It was determined that the patient's bite could be elevated by 2 mm. Consequently, the decision was made to place crowns on teeth 16, 26, 27, 36, and 46. In this patient, due to the atrophy of the maxilla and the necessity to increase the vertical dimension, an implant-supported hybrid prosthesis in relation to 11, 12, 13, 14, 15, 21, 22, 23, 24, and 25 was recommended as it is lightweighted. Removal of the upper implant-supported faulty prosthesis was done, and healing screws were placed. Informed consent was taken from the patient prior to the commencement of the prosthetic phase. 

**Figure 3:**
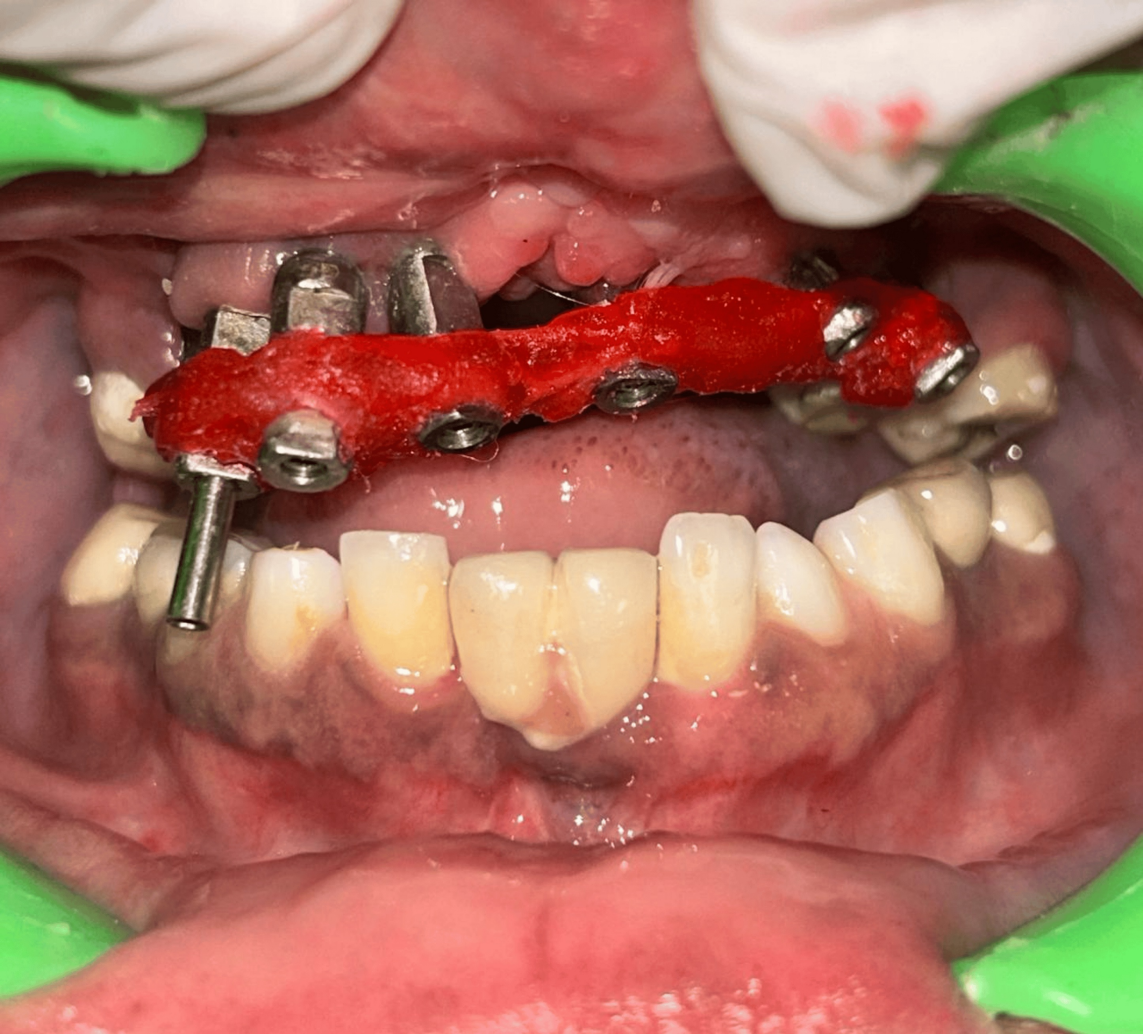
Verification jig tightened intraorally before the final impression

**Figure 4:**
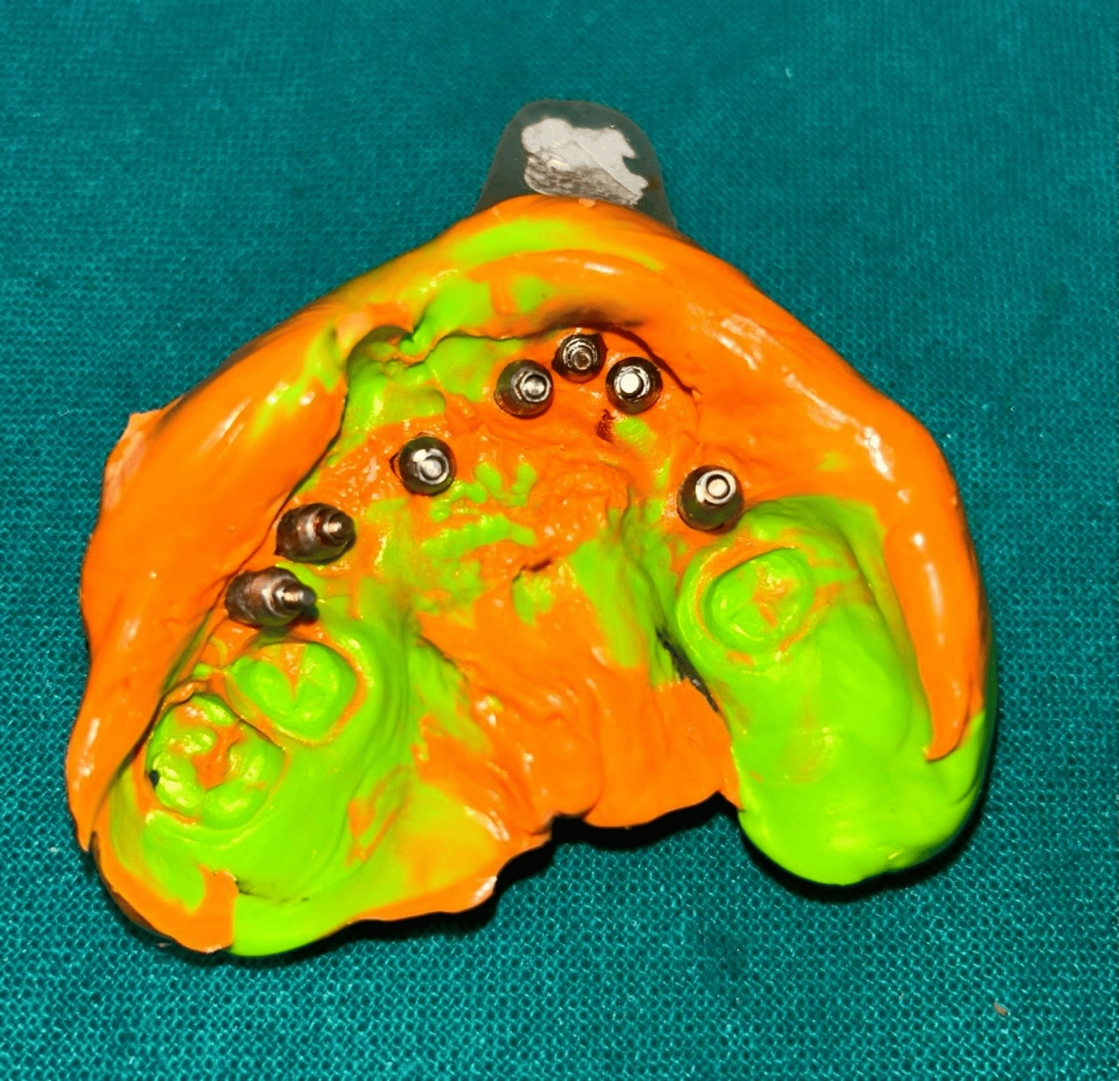
Final impression

First of all, an alginate impression made and modelled was retrieved. A custom impression tray was fabricated on the model made by alginate impression. On this custom tray, holes are made according to implants' position for the open tray impression technique. Before the final impression, the gingival former was unscrewed, and impression posts were tightened over the implants. The open tray impression technique was used for the final impression. Splinting of impression posts was done with the help of dental floss and pattern resin. Implant analogues were attached to the copings, the soft tissue was replicated in the impression using a soft tissue mould material, and the definitive cast was poured with a type 3 dental stone. In the lab over a dental stone model, a splinted pattern resin jig was made on an impression post and sectioned for a jig trial. All sectioned parts of the jig were joined intraorally and checked for passive fit. A jig trial was done, the final impression was made (Figure 3, Figure 4), and the master cast was poured in a similar manner as described above.

**Figure 5:**
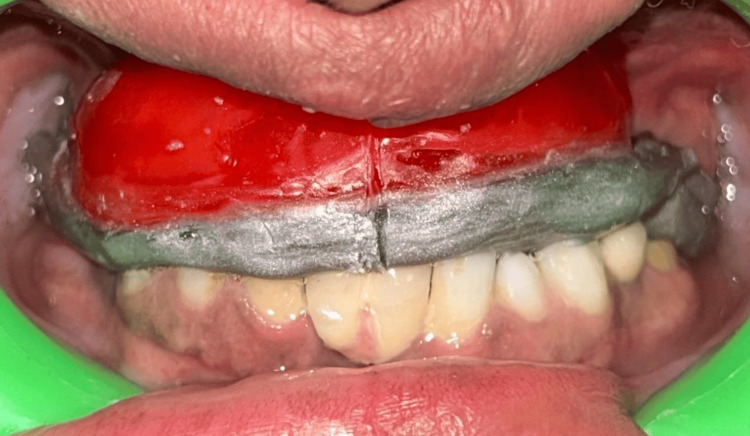
Jaw record

The metal framework, created in the dental laboratory, was evaluated intraorally in the patient's mouth to ensure its fit and passivity. The record base was fabricated over the metal framework, and modelling wax was used for the fabrication of occlusal rims. The occlusal vertical dimension was established, and the patient was asked to close in centric relation (Figure 5). The centric relation record was taken out of the patient's mouth and mounted on the semi-adjustable articulator. Teeth arrangement was done on the articulator, followed by a try-in in the patient's mouth. Once the patient was satisfied with the esthetics and phonetics, the prosthesis was sent for acrylization.

**Figure 6:**
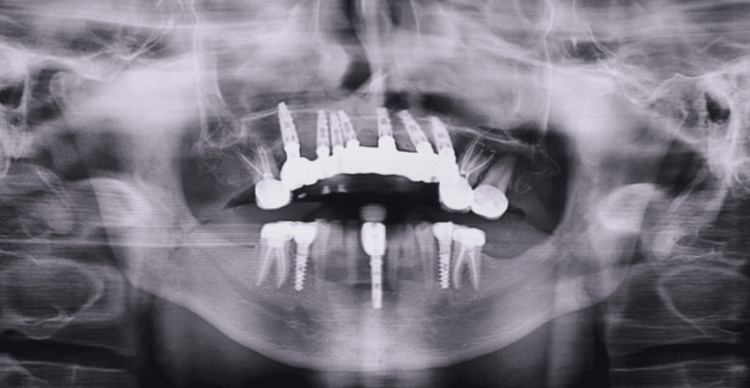
Postoperative radiograph (OPG) OPG: orthopantomography

**Figure 7:**
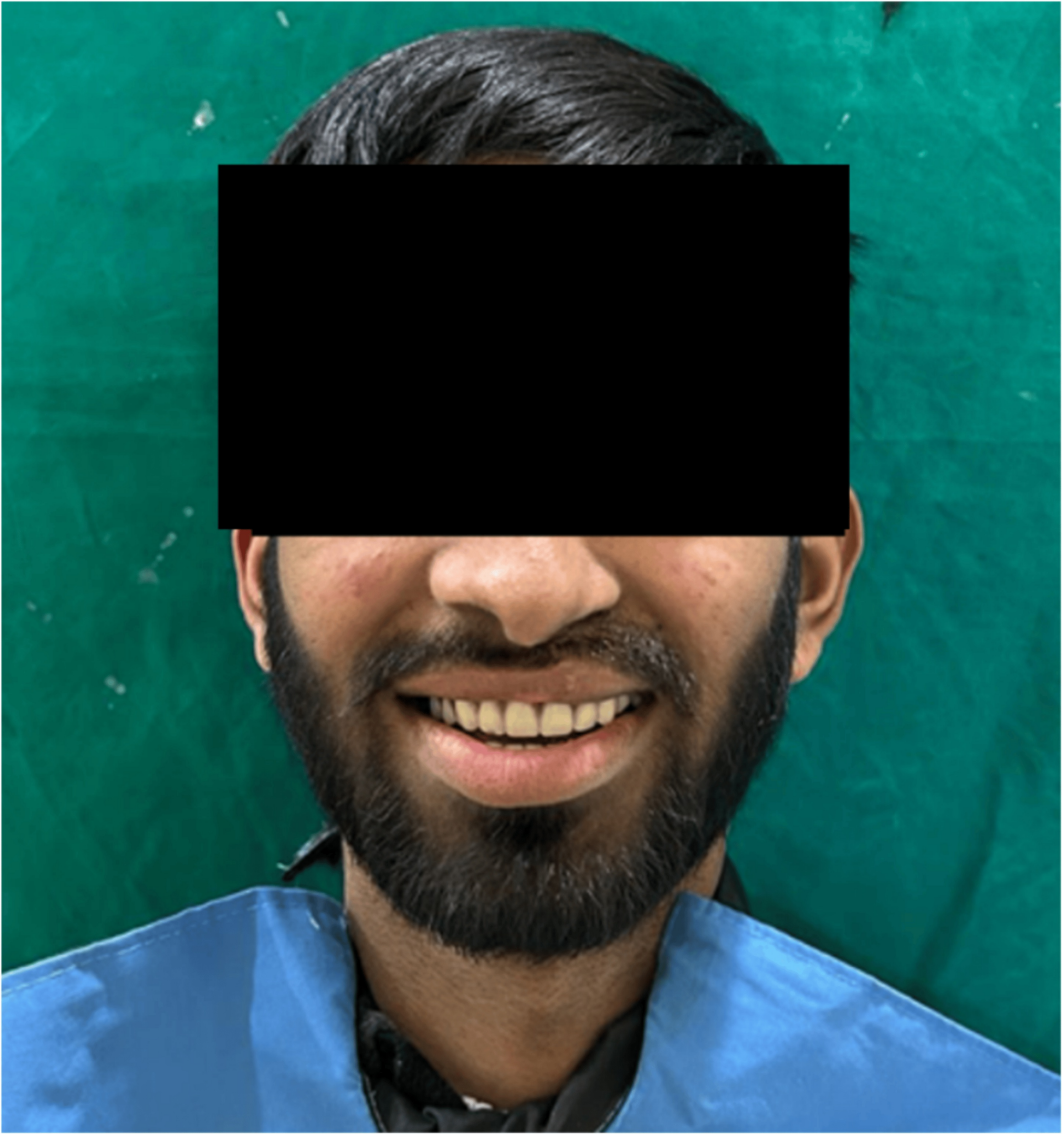
Postoperative front view

Once the acrylized prosthesis is received, the healing cap is removed, and the prosthesis is screwed in and torqued according to the manufacturer's recommendation. The occlusal scheme implemented was that of group function occlusion and then final occlusal adjustment was done using an articulating paper and the screw access openings were initially covered with polytetrafluoroethylene strips and subsequently filled with light-cured flowable composite resin (Figure 6 and Figure 7).

After receiving his prostheses, the patient was provided with instructions on usage and maintenance. He was shown how to use dental floss (ICPA, Younifloss, India) and cordless water flosser (Water Pik, Fort Collins, CO, USA) for maintaining hygiene beneath the restoration, and a follow-up appointment was scheduled after 24 hours for minor adjustments [3].

## Discussion

Thorough planning in dental implantology involves identifying any pre-existing clinical challenges before treatment and anticipating the expected outcomes beforehand [1]. Esthetic case planning necessitates a unique diagnostic approach, encompassing additional factors like smile line, lip line, and lip contour. Moreover, an adequate inter-arch distance is required for a successful prosthetic restoration. In the abovementioned case, it was measured from the implant platform to the occlusal table of opposite arch teeth. For hybrid prosthesis, inter-arch space involves abutment and cast metal framework with acrylic resin and acrylic teeth, greatly affecting the selection of restoration. For maxillary fixed implant-supported prostheses, at least 12-15 mm of space is recommended [2]. If there is ample inter-arch space available, a hybrid prosthesis is typically suggested [4]. In the current case report, it was decided to fabricate an implant-supported hybrid prosthesis for the patient due to the 32 mm intra-arch distance.

Revitalization of a diminished jawbone for dental implant insertion typically involves the placement of the implant following the healing of the graft [5,6]. During the production of a hybrid prosthesis supported by an implant, achieving the framework's passive fit is crucial. Bone loss around the implant, screw loosening, and fracture of abutments and fixtures can occur if the prosthesis does not exhibit a proper passive fit [2].

In this case report, the accuracy of the final model was checked by making an index over it and trying it by screwing it into the implants. The passive fit of the metal framework was verified intraorally to ensure the best outcome [7]. During the follow-up period, no issues such as failure of the implant, soft tissue inflammation around implants, fracture of the framework, fracture of the screw, and screw loosening were seen. No difficulty in maintaining oral hygiene was observed in the patient [8]. When creating an implant-supported fixed complete prosthesis, it's crucial to consider the framework material. Frameworks can be crafted from a range of materials such as base metal alloys, titanium alloys or noble metals, polyetheretherketone (PEEK) material, or zirconia. In these days, zirconia is one of the good options for framework fabrication [8].

Egilmez et al. concluded in their review article that an implant-supported hybrid prosthesis improves the speech, esthetics, and masticatory ability of an edentulous patient [1].

Pjetursson et al. in their systematic review concluded that implant-supported FDPs are a safe and predictable treatment method with high survival rates. However, biological and technical complications were frequent (33.6%). To minimize the incidence of complications, dental professionals should make a great effort in choosing reliable components and materials for implant-supported FDPs, and the patients should be placed in a well-structured maintenance system after treatment [5].

Nonetheless, a reported complication for bilayered ceramic restorations is the fracture and chipping of veneering porcelain [9]. The prostheses in this clinical report were constructed with base metal alloys for their frameworks. The selection of framework material took into account factors such as cost and the potential need for sectioning and soldering to achieve a passive fit. Moreover, research has shown that employing a rigid material can decrease the bending moment on the framework, with cobalt-chromium frameworks exhibiting the lowest strain on implants due to their precise fit [10]. Implant-supported hybrid prostheses can yield satisfactory outcomes in situations where esthetic and functional demands are high and challenging. For instance, when there's increased intra-arch space post-conventional implant replacements, the dentist must devise an alternative treatment approach that is most suitable for the specific condition [2]. The creation of an implant-supported hybrid prosthesis greatly improved the patient's approval of the prosthetic treatment plan and restoration [10]. Another crucial aspect to consider is maintaining both the prosthetic rehabilitation and the implants by providing adequate structural support. Routine checks every six or 12 months are advised to prevent issues related to implant failure. Additionally, it is recommended to measure radiographic peri-implant marginal bone loss during follow-up. In line with earlier guidelines, clinical evaluations were performed at one, two, six, and 12 months after prosthesis placement, with subsequent annual clinical assessment with radiographs [11].

## Conclusions

This case report describes the esthetic and masticatory improvements obtained through the use of an implant-supported hybrid prosthesis. The study concluded that this prosthesis type can deliver satisfactory outcomes in patients who have undergone dental implant placement, provided there is careful treatment planning. The authors also emphasize the importance of evaluating patients not only from a surgical standpoint but also from a prosthodontic perspective.

## References

[1] Egilmez F, Ergun G, Cekic-Nagas I, Bozkaya S (2015). Implant-supported hybrid prosthesis: conventional treatment method for borderline cases. Eur J Dent.

[2] El Askary AE (2008). Fundamentals of esthetic implant dentistry. Ames, Iowa, USA: Munksgaard, Blackwell.

[3] Misch CE (2008). Contemporary implant dentistry. https://search.worldcat.org/title/190612134.

[4] Thalji G, Bryington M, De Kok IJ, Cooper LF (2014). Prosthodontic management of implant therapy. Dent Clin North Am.

[5] Pjetursson BE, Thoma D, Jung R, Zwahlen M, Zembic A (2012). A systematic review of the survival and complication rates of implant-supported fixed dental prostheses (FDPs) after a mean observation period of at least 5 years. Clin Oral Implants Res.

[6] Tourah A, Moshaverinia A, Chee WW (2014). Mandibular implant-supported fixed dental prosthesis with a modified design: a clinical report. J Prosthet Dent.

[7] Dym H, Pierse J (2011). Advanced techniques in bone grafting procedures. Dent Clin North Am.

[8] Beagle JR (2013). Surgical essentials of immediate implant dentistry. Oxford, UK: Wiley-Blackwell.

[9] Sertgöz A, Güvener S (1996). Finite element analysis of the effect of cantilever and implant length on stress distribution in an implant-supported fixed prosthesis. J Prosthet Dent.

[10] Priest G, Smith J, Wilson MG (2014). Implant survival and prosthetic complications of mandibular metal-acrylic resin implant complete fixed dental prostheses. J Prosthet Dent.

[11] Law C, Bennani V, Lyons K, Swain M (2014). Influence of implant framework and mandibular flexure on the strain distribution on a Kennedy class II mandible restored with a long-span implant fixed restoration: a pilot study. J Prosthet Dent.

